# Computed Tomography Imaging Characteristics of Histologically Confirmed Papillary Renal Cell Carcinoma—Implications for Ancillary Imaging

**DOI:** 10.15586/jkcvhl.2019.124

**Published:** 2019-12-30

**Authors:** Jeffrey B. Walker, Justin Loloi, Alexander Birk, Jay D. Raman

**Affiliations:** Division of Urology, Penn State Health Milton S. Hershey Medical Center, Hershey, PA, USA

**Keywords:** CT, histology, Hounsfield, kidney, papillary, RCC

## Abstract

Low-attenuation renal lesions on non-contrast computed tomography (CT) are often considered to be benign cysts without need for further imaging. However, the papillary subtype of renal cell carcinoma (RCC) may have similar radiographic characteristics. A single-center retrospective review was therefore performed to identify extirpated papillary RCC (pRCC) specimens with correlation made to preoperative tumor imaging characteristics. A total of 108 pRCC specimens were identified of which 84 (27 type I, 17 type 2, 40 unspecified) had CT imaging available for review. Non-contrast CT was available for 73 tumors with 16 (22%) demonstrating Hounsfield units (HU) measurements fewer than 20 at baseline without differences between papillary subtypes. Mean attenuation following contrast administration was similar between papillary subtypes (45 HU for type 1 pRCC and 49 HU for type 2). This study highlights that pathologically proven pRCC is a heterogeneous entity in terms of density on preoperative CT imaging. A non-contrast CT scan with HU fewer than 20 may not be an adequate evaluation for incidental renal masses, as over 1 in 5 pRCCs demonstrate lower attenuation than this cutoff. Further study is needed to identify the appropriate role of ancillary imaging in the workup of seemingly benign-appearing renal lesions.

## Introduction

Renal lesions are common incidental findings on computed tomography, seen in 14% of unenhanced colonographic computed tomography (CT) (1). The attenuation of normal renal parenchyma on unenhanced CT typically ranges from 30 to 40 Hounsfield units (HU). Despite a broad differential diagnosis of both benign and malignant entities, a high density lesion measuring 40–70 HU is often considered to be a solid renal neoplasm with the majority having a malignant diagnosis (2). In the management of an incidental renal mass on unenhanced CT, homogeneous lesions with low attenuation, typically defined as fewer than 20 HU, are frequently considered to be cystic lesions with low likelihood of being renal cell carcinoma (RCC) (1, 3). Thus, current guidelines recommend that a renal mass measuring fewer than 20 HU on non-contrast CT does not require further evaluation (4).

With respect to RCC, the papillary histological subtype accounts for approximately 10–15% of all RCCs and typically portends a better prognosis when compared to clear cell carcinomas. Papillary RCC (pRCC) may be classified as either a type 1 or type 2 lesion (5). In terms of cytogenetic and molecular tumor profiling, when compared to type 1 lesions, type 2 pRCC tumors show a greater expression of vascular endothelial growth factor (VEGF) in the tumor epithelium and endothelium (6). Consequently, type 2 pRCC tumors are commonly associated with higher T stage, nodal and distant metastasis, higher grade, and higher degree of necrosis. These aggressive clinicopathological features along with the significantly higher microvascular invasion observed in type 2 lesions are also associated with worse outcomes when compared with type I (7, 8).

pRCC may present as a low-attenuation tumor on CT due to differences in the intratumoral vascularity and microvessel density. These differences enable clinicians to distinguish it from other types of RCC. However, there is a paucity of data directly delineating the frequency with which pRCC presents as a low-attenuation mass on non-contrast CT. This study aims to describe the CT characteristics of pRCC and to compare the differences between type 1 and type 2 pRCC, as well as suggests the role of ancillary imaging to better address this imaging conundrum.

## Materials and Methods

Radical and partial nephrectomies performed between July 2007 and July 2017 at a single tertiary care academic medical center were retrospectively reviewed following IRB approval. Pathology reports were reviewed for all patients with pT1 or pT2 disease to identify cases with a histologically confirmed diagnosis of pRCC. Patients with preoperative renal CT imaging within 6 months of surgery were included for analysis. Exclusion criteria included the presence of multiple RCC types on pathologic specimen, inability to correlate pathology report with preoperative imaging when multiple lesions were present, and unavailability of preoperative CT scans for independent review.

In all cases, four CT scan phases were analyzed to obtain renal tumor characteristics—non-contrast, corticomedullary, nephrographic, and pyelographic. Approximate times following intravenous contrast for the contrast phases were 30 s, 100 s, and 10 min, respectively, although there were minor variations. CT phases were available in various combinations as preoperative imaging selection was patient-dependent. For each phase, the renal tumor was assessed for maximum dimension in millimeters in all projections. The tumor was then assessed for density in the axial projection by selecting 6 evenly spaced axial regions and using the imaging software to calculate HU. These six values were averaged and recorded. This method has been utilized previously in the literature and represents a real-world approach clinicians employ when reviewing CT imaging. Preliminary comparisons between using six evenly spaced regions versus all regions confirmed the validity of this approach. Tumors were grouped in increments of 10 HU to determine the frequency of observed preoperative imaging density.

Differences in papillary subtype characteristics were compared using two-tailed t-tests.

## Results

Between July 2007 and July 2017, a total of 57 radical nephrectomies and 67 partial nephrectomies were performed at our institution for a combined 124 pathologically confirmed cases of papillary RCC (Table 1); 16 of these specimens contained other additional renal cell carcinoma types and were excluded. Of the 108 remaining patients, 84 (70% women, average age 62 years) had preoperative CT imaging available for review. Of these, 27 were reported on pathology to represent type 1 papillary RCC, 17 were type 2, and 40 were unspecified. The median largest tumor dimension was 39.5 mm, ranging from 18 to 170 mm.

**Table 1:** Patient and tumor characteristics.

Total patients with pRCC	124		
Radical nephrectomies	57		
Partial nephrectomies	67		
Patients with exclusively pRCC	108		
Patients with CT available	84		
Patient age (mean, range)	62		20 to 94
Female gender (#, %)	59		70%
Type 1 pRCC (#, %)	27		32%
Type 2 pRCC (#, %)	17		20%
Type unspecified pRCC (#, %)	40		48%
Tumor size (mm, range)	39.5		18 to 170
Noncontrast HU<10 (#, %)	3		4%
Noncontrast HU<20 (#, %)	15		21%
Noncontrast HU<30 (#, %)	45		62%
Noncontrast HU (mean, SD, range)	27	10	−6 to 50
30-s contrast HU (mean, SD, range)	41	17	7 to 91
100-s contrast HU (mean, SD, range)	46	21	8 to 97
10-min contrast HU (mean, SD, range)	47	17	20 to 101

pRCC, Papillary renal cell carcinoma; CT, computed tomography; HU, Hounsfield units; SD, standard deviation.

**Figure 1:**
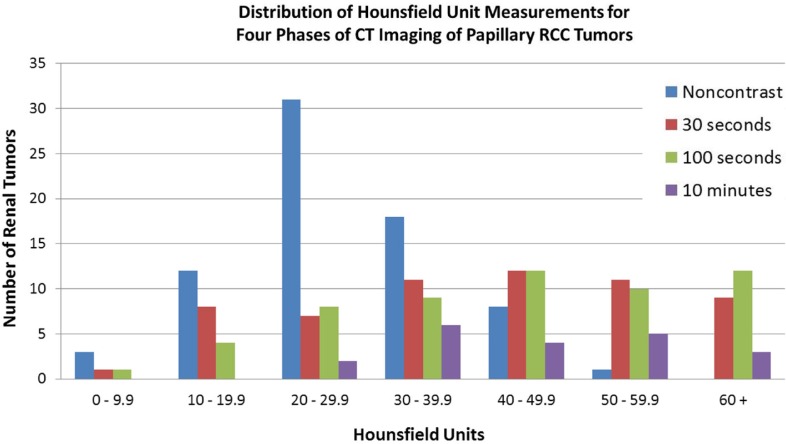
Distribution of Hounsfield unit measurements for four phases of CT imaging of papillary RCC tumors.

HU measurements on preoperative CT scan varied widely within all four imaging phases analyzed (Figure 1). Mean HU at 30 s, 100 s, and 10 min were 41 (standard deviation [SD] 17, range 7 to 91), 46 (SD 21, range 8 to 97), and 47 (SD 17, range 20 to 101). The mean non-contrast attenuation was 27 HU (SD 10, range −6 to 50) for the 73 tumors for which a non-contrast phase was available. Of these, three (4%) had HU measuring less than 10; 15 (21%) had HU measuring less than 20; and 45 (62%) had HU measuring less than 30. Nine (60%) of the 15 tumors with HU measuring fewer than 20 were clinical stage T1 or T2; five were papillary type 1; three were papillary type 2; and seven were unspecified.

On non-contrast imaging, mean HU was 25 for papillary type 1 tumors and 27 for type 2 tumors. There was no significant difference on two-tailed t-test in attenuation between type 1 and type 2 tumors (P=0.67). At the 30-s contrast-enhanced phase, mean HU was 37 for type 1 tumors and 49 for type 2 tumors; this difference was also not significant (P=0.06).

## Discussion

We present data describing the frequent occurrence of low-attenuation pRCC on non-contrast CT. We found that over 20% of pRCC lesions measured fewer than 20 HU. These results suggest that a renal mass with non-contrast CT demonstrating fewer than 20 HU may not be an adequate evaluation for incidental renal masses to forego further workup.

CT remains the first-line imaging modality for the characterization of renal masses (9). A proportion of RCC, more specifically pRCC, are hypo-enhancing and may show indeterminate range (10–20 HU) or even absent attenuation (fewer than 10 HU) on multi-phase CT given their hypovascular and homogenous nature (10). pRCC characteristically demonstrates gradual progressive enhancement. In a retrospective study consisting of 27 pRCCs, Dilauro et al. compared contrast-enhanced CT with magnetic resonance imaging (MRI) for the diagnosis of pRCC. They demonstrated that only a small number of pRCCs have indeterminate enhancement on CT when a renal protocol is used. Specifically, on unenhanced CT, attenuation of pRCCs was similar to that of hemorrhagic or proteinaceous cysts, with a mean attenuation of 35.7 HU (range 19 to 66) (11). Schieda et al. determined the mean attenuation for 27 papillary tumors to be approximately 35 HU on unenhanced CT, with none having attenuation between −10 and 20 (12). These observations contrast with our study, which found that greater than 20% of papillary masses measured fewer than 20 HU on non-contrast CT. This finding highlights the potential of missing clinically meaningful tumors with the current practice of foregoing workup for a hypoenhancing lesion.

The reason for the discrepancy between our findings and previous literature is unclear. One possible explanation may be related to the retrospective single-institution nature of the study. If our center’s threshold for recommending extirpative therapy differs from elsewhere, we may be capturing more low-density papillary tumors in our data set. Additionally, as CT technology, and other modalities such as ultrasound, continues to improve, more and more subtle lesions may be picked up, leading to their surgical removal. Finally, our sample size is several times greater than that of previous studies.

Types 1 and 2 pRCC can be distinguished pathologically. Type 1 tumors are characterized by a single layer of small cells with scant cytoplasm, whereas type 2 lesions contain high nuclear-grade cells with abundant eosinophilic cytoplasm, coinciding with considerably poorer prognostic outcomes (13). However, from an imaging standpoint, we found similarity between type 1 and 2 pRCC, consistent with prior study (14).

There are no large studies describing specific distinguishing features between types 1 and 2 pRCC. However, Yamada et al. conducted a retrospective review of pRCC patients, aiming to elaborate the unique CT features for each type of pRCC. They showed that type 1 tumors have more distinct margins and homogenous density than type 2 tumors, while both tumors were noted to show minimal enhancement. Specifically, the mean attenuation on non-contrast CT did not differ significantly between the two types, with a recorded measure of 34.6 and 38.4 HU for type 1 and 2 tumors, respectively (15). Compared to type 1 tumors, type 2 tumors showed slightly higher mean relative enhancement ratios during the corticomedullary and nephrogenic phase, although these changes were not significant. In our study, the imaging characteristics for types 1 and 2 tumors were comparable, as we did not find a statistically significant mean attenuation on noncontrast imaging or within contrast phases, consistent with the literature to date.

With improvements in diagnostic imaging modalities, the future directions in the evaluation of the renal mass are trending towards the use of MRI. This is thought to be attributable in part to an increased concern with radiation exposure associated with CT scans (16). In today’s practice, the American College of Radiology rates MRI comparable to CT for the evaluation of indeterminate renal masses (17). Some studies suggest that MRI is the best modality for providing information to diagnose RCC subtypes as it enables high-quality histopathological characterization of renal masses (18). Specifically, for pRCC, Chiarello et al. conducted a systematic review and meta-analysis to delineate the diagnostic performance of MRI in differentiating pRCC from other masses (19). Their study compared 275 pRCC lesions with 758 other renal masses. The results suggested that MRI has a strong sensitivity and specificity for quantitative enhancement for differentiating pRCC from other tumors, particularly during the corticomedullary phase (19). In our study, 31 of the 108 (29%) pRCC tumors had preoperative MRI imaging available, with 24 of these 31 cases (77%) in the more contemporary time period. Thus, although our study is not focused on the implications of MRI in the evaluation of RCC, it is clear that MRI is increasingly playing a more significant role in RCC imaging at our institution.

Our study findings are limited due to the retrospective nature of the study. All patients did not undergo a standardized imaging workup prior to surgery, and thus, not all CT imaging phases are available for each lesion. As the papillary subtype represents only about 20% of all RCC, our sample size is limited. Furthermore, pathologic diagnoses may vary among pathologists, and not all specimens were specified as type 1 or type 2.

## Conclusion

PRCC is a heterogeneous entity. Current clinical practice entails foregoing further workup for renal lesions measuring fewer than 20 HU on noncontrast CT. This study demonstrates that over 20% of pRCC measures at fewer than 20 HU, suggesting that this criterion may not yield adequate evaluation for an incidental renal mass. Further research will help better define the role of ancillary imaging.

## References

[1] O'Connor SD, Pickhardt PJ, Kim DH, Oliva MR, Silverman SG Incidental finding of renal masses at unenhanced CT: Prevalence and analysis of features for guiding management. AJR Am J Roentgenol. 2011;197:139–45. 10.2214/AJR.10.5920 21701022

[2] Kang SK, Huang WC, Pandharipande PV, Chandarana H Solid renal masses: What the numbers tell us. AJR Am J Roentgenol. 2014;202:1196–206. 10.2214/AJR.14.12502 24848816 PMC4174582

[3] Pooler BD, Pickhardt PJ, O'Connor SD, Bruce RJ, Patel SR, Nakada SY Renal cell carcinoma: Attenuation values on unenhanced CT. AJR Am J Roentgenol. 2012;198:1115–20. 10.2214/AJR.11.7587 22528901

[4] Herts BR, Silverman SG, Hindman NM, Uzzo RG, Hartman RP, Israel GM, et al. Management of the incidental renal mass on CT: A white paper of the ACR Incidental Findings Committee. J Am Coll Radiol. 2018;15:264–73. 10.1016/j.jacr.2017.04.028 28651987

[5] Egbert ND, Caoili EM, Cohan RH, Davenport MS, Francis IR, Kunju LP, et al. Differentiation of papillary renal cell carcinoma subtypes on CT and MRI. AJR Am J Roentgenol. 2013;201:347–55. 10.2214/AJR.12.9451 23883215

[6] Klatte T, Pantuck AJ, Said JW, Seligson DB, Rao NP, LaRochelle JC, et al. Cytogenetic and molecular tumor profiling for type 1 and type 2 papillary renal cell carcinoma. Clin Cancer Res. 2009;15:1162–9. 10.1158/1078-0432.CCR-08-1229 19228721

[7] Pignot G, Elie C, Conquy S, Vieillefond A, Flam T, Zerbib M, et al. Survival analysis of 130 patients with papillary renal cell carcinoma: Prognostic utility of type 1 and type 2 subclassification. Urology. 2007;69:230–5. 10.1016/j.urology.2006.09.052 17275070

[8] Delahunt B, Eble JN, McCredie MR, Bethwaite PB, Stewart JH, Bilous AM Morphologic typing of papillary renal cell carcinoma: Comparison of growth kinetics and patient survival in 66 cases. Hum Pathol. 2001;32:590–5. 10.1053/hupa.2001.24984 11431713

[9] Krishna S, Murray CA, McInnes MD, Chatelain R, Siddaiah M, Al-Dandan O, et al. CT imaging of solid renal masses: Pitfalls and solutions. Clin Radiol. 2017;72:708–21. 10.1016/j.crad.2017.05.003 28592361

[10] Herts BR, Coll DM, Novick AC, Obuchowski N, Linnell G, Wirth SL, et al. Enhancement characteristics of papillary renal neoplasms revealed on triphasic helical CT of the kidneys. AJR Am J Roentgenol. 2002;178:367–72. 10.2214/ajr.178.2.1780367 11804895

[11] Dilauro M, Quon M, McInnes MD, Vakili M, Chung A, Flood TA, et al. Comparison of contrast-enhanced multiphase renal protocol CT versus MRI for diagnosis of papillary renal cell carcinoma. AJR Am J Roentgenol. 2016;206:319–25. 10.2214/AJR.15.14932 26797358

[12] Schieda N, Vakili M, Dilauro M, Hodgdon T, Flood TA, Shabana WM Solid renal cell carcinoma measuring water attenuation (-10 to 20 HU) on unenhanced CT. AJR Am J Roentgenol. 2015;205:1215–21. 10.2214/AJR.15.14554 26587928

[13] Prasad SR, Humphrey PA, Catena JR, Narra VR, Srigley JR, Cortez AD, et al. Common and uncommon histologic subtypes of renal cell carcinoma: Imaging spectrum with pathologic correlation. Radiographics. 2006;26:1795–806;discussion 1806–710. 10.1148/rg.266065010 17102051

[14] Vikram R, Ng CS, Tamboli P, Tannir NM, Jonasch E, Matin SF, et al. Papillary renal cell carcinoma: Radiologic-pathologic correlation and spectrum of disease. Radiographics. 2009;29:741–54;discussion 755–47. 10.1148/rg.293085190 19448113

[15] Yamada T, Endo M, Tsuboi M, Matsuhashi T, Takase K, Higano S, et al. Differentiation of pathologic subtypes of papillary renal cell carcinoma on CT. AJR Am J Roentgenol. 2008;191:1559–63. 10.2214/AJR.07.3181 18941101

[16] Sankineni S, Brown A, Cieciera M, Choyke PL, Turkbey B Imaging of renal cell carcinoma. Urol Oncol. 2016;34:147–55. 10.1016/j.urolonc.2015.05.020 26094171

[17] American College of Radiology ACR Appropriateness Criteria^®^.

[18] Gurel S, Narra V, Elsayes KM, Siegel CL, Chen ZE, Brown JJ Subtypes of renal cell carcinoma: MRI and pathological features. Diagn Interv Radiol. 2013;19:304–11. 10.5152/dir.2013.147 23439256

[19] Chiarello MA, Mali RD, Kang SK Diagnostic accuracy of MRI for detection of papillary renal cell carcinoma: A systematic review and meta-analysis. AJR Am J Roentgenol. 2018;211:812–21. 10.2214/AJR.17.19462 30063398 PMC6440798

